# Borderline Personality Disorder in the Andalusian General Population: Prevalence, Sociodemographic Correlates, and Psychiatric Comorbidities

**DOI:** 10.1192/j.eurpsy.2026.11912

**Published:** 2026-07-31

**Authors:** I. Ramos-Suarez, M. Guerrero-Jiménez, B. Gutiérrez

**Affiliations:** 1 Psychiatry, Hospital Universitario Clínico San Cecilio; 2 Department of Psychiatry, Faculty of Medicine, University of Granada, Granada, Spain

## Abstract

**Introduction:**

Borderline Personality Disorder (BPD) is a severe and often underdiagnosed condition associated with emotional instability, impulsivity, and intense interpersonal difficulties. It is also linked to high rates of suicide attempts, psychiatric comorbidities, and functional impairment. While BPD has been extensively studied in clinical populations, there is limited epidemiological data from representative community samples in Southern Europe. Andalusia, the most populated region in Spain, lacks studies estimating the prevalence and associated factors of BPD in the general population.

**Objectives:**

This study aims to:

1. Estimate the point prevalence of Borderline Personality Disorder in the general adult population of Andalusia using DSM-IV-based diagnostic criteria and validated clinical tools.
2. Identify sociodemographic factors independently associated with the presence of BPD, including age, sex, marital status, education level, and income.
3. Explore the psychiatric comorbidity profile of individuals with BPD, focusing on mood disorders, anxiety disorders, substance use disorders, and suicidal behavior.

**Methods:**

Borderline Personality Disorder (BPD) is a severe and often underdiagnosed condition associated with emotional instability, impulsivity, and intense interpersonal difficulties. It is also linked to high rates of suicide attempts, psychiatric comorbidities, and functional impairment. While BPD has been extensively studied in clinical populations, there is limited epidemiological data from representative community samples in Southern Europe. Andalusia, the most populated region in Spain, lacks studies estimating the prevalence and associated factors of BPD in the general population.

**Results:**

Borderline Personality Disorder (BPD) is a severe and often underdiagnosed condition associated with emotional instability, impulsivity, and intense interpersonal difficulties. It is also linked to high rates of suicide attempts, psychiatric comorbidities, and functional impairment. While BPD has been extensively studied in clinical populations, there is limited epidemiological data from representative community samples in Southern Europe. Andalusia, the most populated region in Spain, lacks studies estimating the prevalence and associated factors of BPD in the general population.

**Conclusions:**

This is the first study to estimate the prevalence and correlates of BPD in a large, representative community sample in Andalusia. The results will provide valuable insights into the burden and distribution of BPD in the general population, informing future mental health policies and targeted interventions in primary care and community settings.

**Disclosure of Interest:**

None Declared

